# Assessment of the Association of Periodontitis and Diabetes Mellitus with Alzheimer’s Disease in a Mouse Model

**DOI:** 10.3390/ijms27177542

**Published:** 2026-08-23

**Authors:** Momoko Nakahara, Kota Kataoka, Takayuki Maruyama, Mohammad Nurhamim, Yixuan Zhang, Daiki Fukuhara, Yoko Uchida-Fukuhara, Md Monirul Islam, Manabu Morita, Takashi Saito, Daisuke Ekuni

**Affiliations:** 1 Department of Preventive Dentistry, Faculty of Medicine, Dentistry and Pharmaceutical Sciences, Okayama University, Okayama 700-8558, Japan; t-maru@md.okayama-u.ac.jp (T.M.); dekuni7@md.okayama-u.ac.jp (D.E.); 2 Department of Preventive Dentistry, Division of Dentistry, Medical Development Field, Okayama University, Okayama 700-8558, Japan; de18017@s.okayama-u.ac.jp; 3 Department of Preventive Dentistry, Graduate School of Medicine Dentistry and Pharmaceutical Sciences, Okayama University, Okayama 700-8558, Japan; dr.nurhamim@gmail.com (M.N.); py466nau@s.okayama-u.ac.jp (Y.Z.); 4 Department of Preventive Dentistry, Dental School, Okayama University, Okayama 700-8558, Japan; de20041@s.okayama-u.ac.jp; 5 Laboratory of Biological Anthropology, Research Center for Integrative Evolutionary Science, The Graduate University for Advanced Studies, SOKENDAI, Hayama 240-0193, Japan; de20006@s.okayama-u.ac.jp; 6 Centre for Policy Studies, University College Cork, T12 K8AF Cork, Ireland; monirul2086@gmail.com; 7 Department of Oral Health Sciences, Faculty of Health Care Sciences, Takarazuka University of Medical and Health Care, Takarazuka 531-0071, Japan; m.morita@tumh.ac.jp; 8 Department of Neuropathology, Graduate School of Medicine, The University of Tokyo, Tokyo 113-0033, Japan; tsaito@m.u-tokyo.ac.jp

**Keywords:** Alzheimer’s disease, periodontitis, diabetes mellitus, microRNA, mRNA

## Abstract

The purpose of the present study was to investigate how periodontitis and diabetes mellitus (DM) are associated with Alzheimer’s disease (AD) through microRNA (miRNA) using AD model mice. The experimental period was 8 weeks. Twenty-four male knock-in mice (B6-AppNL-G-F/NL-G-F/J) were divided into four groups: control group fed a normal diet (C), DM group fed a high-fat/sucrose diet (DM), periodontitis (P) group, and DM + periodontitis (DM+P) group. Memory performance was compared using the Y-maze test. Next-generation sequencing was performed on brain samples, and fold changes in miRNA expression were calculated by comparing the DM+P and C groups. Integrated miRNA–mRNA analysis identified putative miRNA-targeted mRNAs, and protein expression of the top candidate gene was assessed. Memory function in the DM+P group was significantly lower than in the C group. Among the seven mRNAs identified by the integrated analysis, Neurod1 showed the greatest decrease in expression, and it was predicted to be regulated by miR-693-3p. Neurod1 protein expression in the hippocampus was significantly lower in the DM+P group than the C group. Our results suggest that the combined exposure to periodontitis and DM was associated with AD-like pathological changes and identified the miR-693-3p/Neurod1 pair as a candidate regulatory axis.

## 1. Introduction

Alzheimer’s disease (AD) is the most common cause of dementia [1,2]. Amyloid-β (Aβ) deposition and its neurotoxicity in areas of the brain, such as the hippocampus, play a causative role in AD [3]. Aβ is generated by aberrant processing of Aβ precursor protein (APP) in the brain during AD [4,5]. Despite significant progress in understanding AD, certain pathophysiological mechanisms remain incompletely understood.

Among the various factors that may contribute to the pathogenesis of AD, periodontitis has emerged as a potential risk factor. Previous systematic reviews have shown a positive association between periodontitis and AD [6,7,8,9]. In a retrospective cohort study, Choi et al. reported that patients with chronic periodontitis are at higher risk of overall dementia and AD than healthy individuals [10]. In a cohort study by Chen et al., patients with 10 years of exposure to chronic periodontitis exhibited a higher risk of developing AD than unexposed patients [11]. Previous studies investigating cognitive performance reported that periodontitis or periodontal pathogens have a negative effect on animal cognitive behavior, such as memory decline and loss of learning and spatial capabilities [6]. Additionally, studies in AD animal models have indicated that periodontal ligatures are significantly associated with cognitive impairment [12]. Systematic reviews have suggested that periodontitis or periodontal bacteria may contribute to neuroinflammation, which may be associated with brain tissue damage and cognitive impairment [6,13,14,15]. However, the pathophysiological mechanisms that link AD and periodontitis remain poorly understood.

Type 2 diabetes mellitus (T2DM) is another important risk factor for AD. T2DM is characterized by hyperglycemia, hyperinsulinemia, insulin resistance, and peripheral inflammation, and it increases the risk of AD [16,17,18,19]. Prospective longitudinal studies have shown that T2DM is associated with an increased risk of AD compared with non-diabetic individuals [20,21,22,23,24,25,26]. Insulin resistance in T2DM is thought to contribute to the neuropathology of AD by increasing inflammation, tau phosphorylation, Aβ deposition, and impeding Aβ clearance [16,27,28]. The peripheral hyperinsulinemia associated with T2DM may contribute to Aβ accumulation through competition between insulin and Aβ for insulin-degrading enzyme [29,30]. Evidence also suggests that brain insulin resistance upregulates APP and β-secretase 1 (BACE1), in turn leading to Aβ formation [29,31]. An animal study using Thy1-C/EBPβ transgenic mice showed that diabetes mellitus (DM)-associated chronic inflammation activates C/EBPβ/AEP signaling, which contributes to the pathogenesis of AD [32]. However, the molecular mechanism underlying the association between DM and AD is not fully understood.

The complexity of the relationship between AD, periodontitis, and T2DM suggests that microRNAs (miRNAs) may function as important regulatory molecules in these pathological processes. miRNAs are single-stranded, noncoding RNAs that play critical roles in post-transcriptional gene regulation [33,34]. These small molecules are known to bind to the 3′-untranslated region of targeted messenger RNAs (mRNAs) via sequence complementarity [35,36,37]. Considerable research has focused on miRNAs known to contribute to various biological and pathological mechanisms for their potential as biomarkers and therapeutics for many diseases, including AD, DM, cancer, and inflammatory diseases such as periodontitis [38,39,40,41,42,43,44,45,46,47]. Although miRNAs are gaining attention as potentially useful therapeutic targets for AD, no miRNA-based therapeutic approaches have been successful in the clinical management of AD; thus, further investigation is needed [41].

Few studies have investigated the potential role of miRNA-mediated regulation in the relationship between periodontitis, DM, and AD using an experimental animal model. In particular, the combined effects of periodontitis and DM on AD have rarely been examined by comprehensively evaluating brain miRNA and mRNA expression. We hypothesized that periodontitis and DM may influence AD through miRNA-mediated regulatory mechanisms and that their combined exposure may be associated with more pronounced AD-related alterations. The purpose of this study was to investigate how periodontitis and DM are associated with AD through miRNAs using AD model mice and to explore candidate miRNA–mRNA regulatory relationships associated with changes observed following combined exposure to periodontitis and DM.

## 2. Results

### 2.1. Comparison of Body Weight

**Figure 1:**
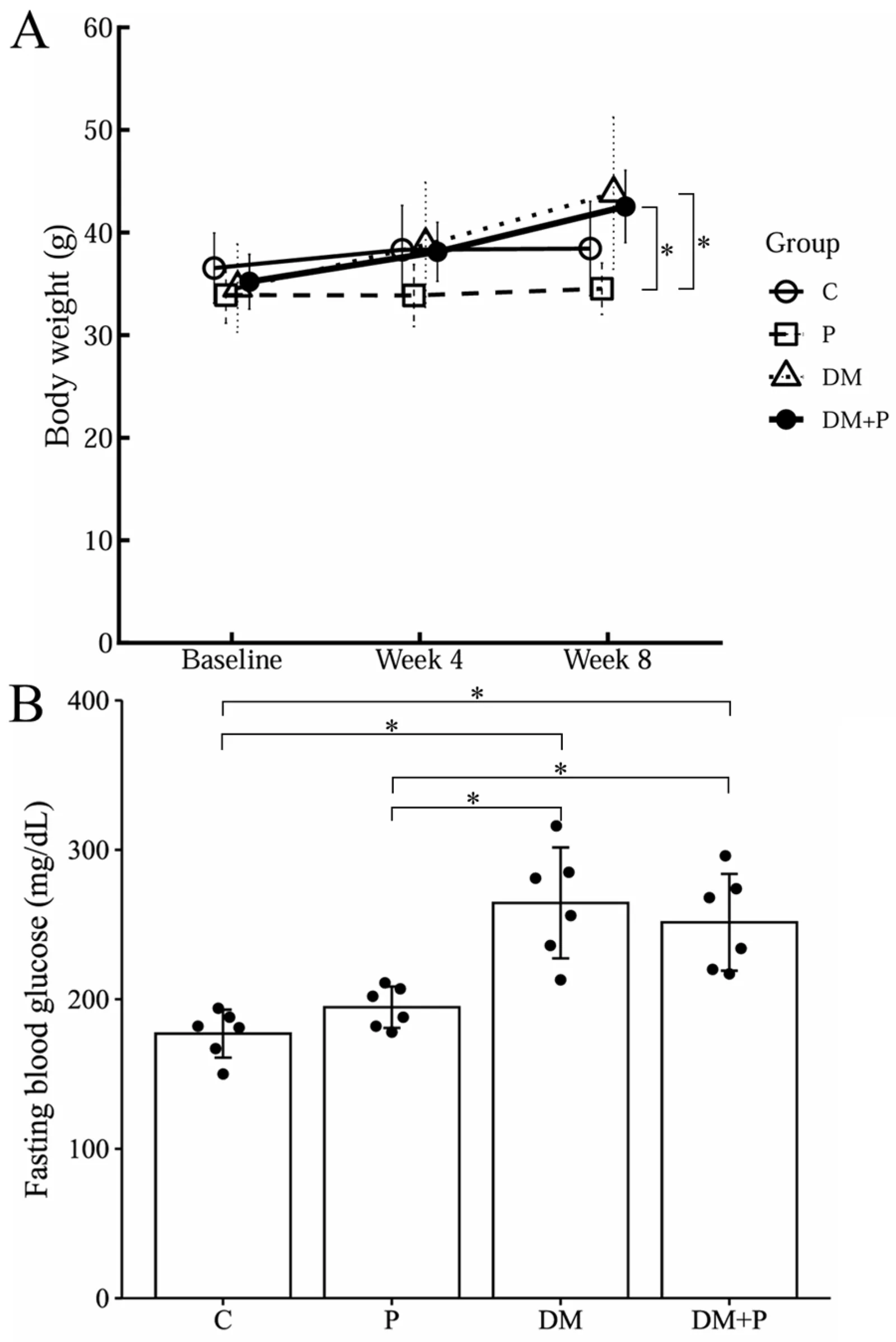
Body weight changes during the experimental period and fasting blood glucose levels at 8 weeks. (**A**) Body weight. * After 8 weeks, body weight was significantly higher in the DM and DM+P groups than in the P group (*p* < 0.05). (**B**) Fasting blood glucose levels after 8 weeks. * Fasting blood glucose levels were significantly higher in the DM and DM+P groups than in the C and P groups (*p* < 0.01). Data are expressed as the mean ± standard deviation (SD) of body weight (g) or fasting blood glucose levels (mg/dL) of mice in the C, P, DM, and DM+P groups (*n* = 6 per group). Statistical significance was determined using the two-way repeated measures analysis of variance (ANOVA) (**A**) and one-way ANOVA (**B**) followed by Tukey’s post hoc test. Abbreviations: C, control; P, periodontitis; DM, diabetes mellitus; DM+P, DM + periodontitis.

In the two-way repeated measures analysis of variance (ANOVA), a significant main effect of time on body weight was observed (*F*(1.365, 27.292) = 55.653, *p* < 0.001, partial *η*^2^ = 0.736), and the interaction between group and time was also significant (*F*(4.094, 27.292) = 10.973, *p* < 0.001, partial *η*^2^ = 0.622). In Tukey’s post hoc test, no significant differences in body weight were observed among the four groups at baseline and after 4 weeks (Figure 1A). After 8 weeks, body weight was significantly higher in the DM (95% CI, 1.452 to 17.182; *p* = 0.017) and DM + periodontitis (DM+P) (95% CI, 0.152 to 15.882; *p* = 0.045) groups than in the periodontitis (P) group. No significant differences were observed between groups control (C) and P, between groups C and DM, between groups C and DM+P, or between groups DM and DM+P. After 8 weeks, fasting blood glucose levels were significantly higher in the DM group than in the C (95% CI, 44.193 to 130.807; *p* < 0.001) and P (95% CI, 26.526 to 113.141; *p* = 0.001) groups in the one-way ANOVA followed by Tukey’s post hoc test (Figure 1B). Fasting blood glucose levels were significantly higher in the DM+P group than in the C (95% CI, 31.193 to 117.807; *p* = 0.001) and P (95% CI, 13.526 to 100.141; *p* = 0.008) groups.

### 2.2. Comparison of Alveolar Bone Loss

**Figure 2:**
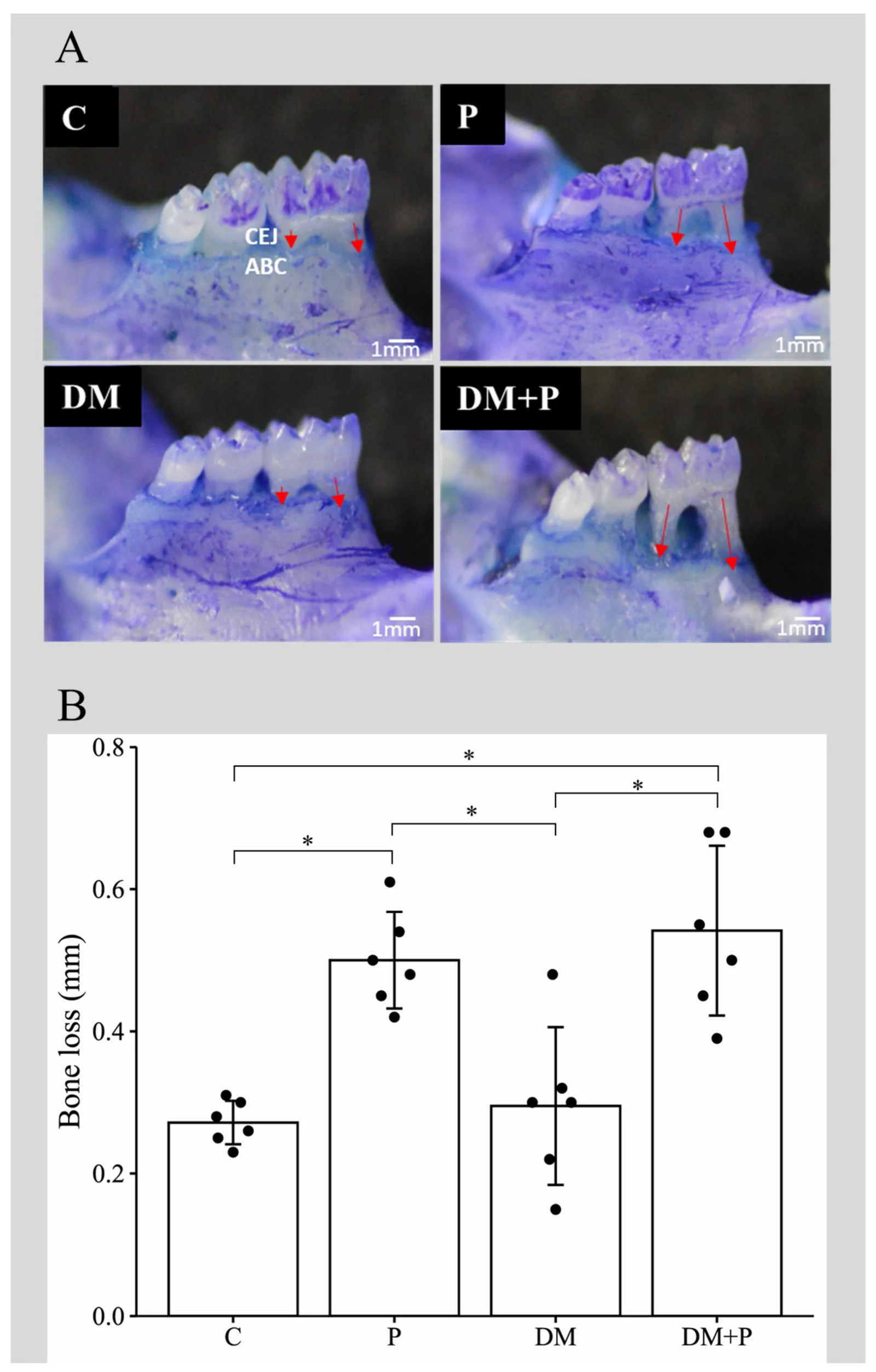
Comparison of alveolar bone loss. (**A**) Representative images of the right mandibular molar regions. Red arrows indicate the distance from the cement–enamel junction (CEJ) to the alveolar bone crest (ABC). (**B**) Comparison of alveolar bone loss among the four groups. * *p* < 0.01. Data are expressed as the mean ± SD of alveolar bone loss (mm) of mice in the C, P, DM, and DM+P groups. Statistical significance was determined using a one-way ANOVA followed by Tukey’s post hoc test.

Alveolar bone loss was significantly greater in the P group than in the C (95% CI, 0.085 to 0.375; *p* = 0.001) and DM (95% CI, 0.061 to 0.352; *p* = 0.004) groups, and alveolar bone loss was also significantly greater in the DM+P group than in the C (95% CI, 0.125 to 0.415; *p* < 0.001) and DM (95% CI, 0.101 to 0.392; *p* = 0.001) groups (Figure 2A,B).

### 2.3. Comparison of the Behavioral Alternation Rate

**Figure 3:**
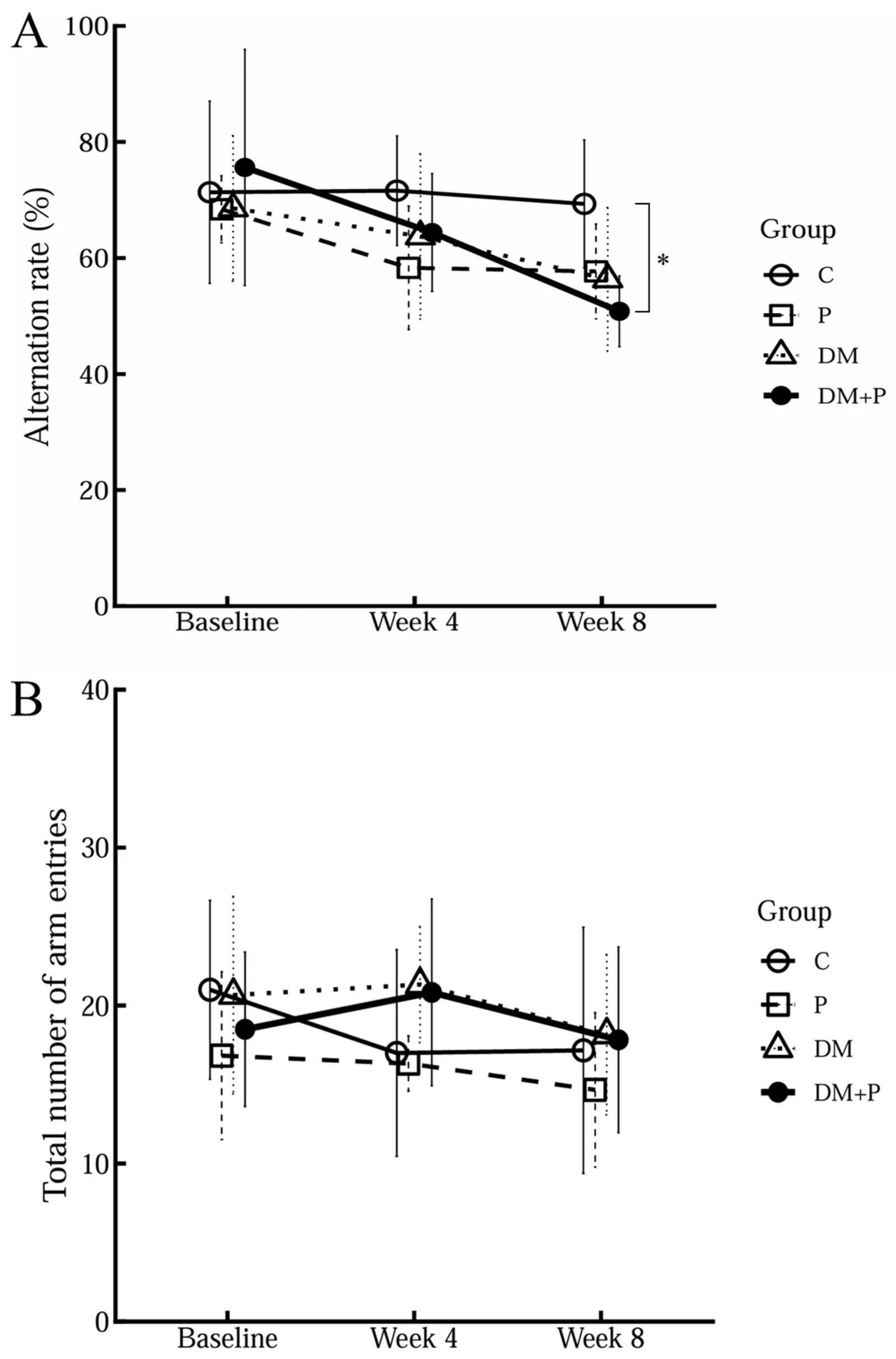
Changes in behavioral alternation rate and total number of arm entries as measured using the Y-maze test during the experimental period. (**A**) Alternation rate (%). * After 8 weeks, the alternation rate was significantly lower in the DM+P group than in the C group (*p* = 0.017). (**B**) Total number of arm entries. No significant differences were observed among the four groups at any time point. Data are expressed as the mean ± SD in mice from the C, P, DM, and DM+P groups. Statistical significance was determined using the two-way repeated measures ANOVA followed by Tukey’s post hoc test.

In the two-way repeated measures ANOVA, a significant main effect of time on the alternation rate was observed (*F*(2,40) = 6.742, *p* = 0.003, partial *η*^2^  =  0.252), whereas the interaction between group and time was not significant. In Tukey’s post hoc test, the alternation rate was significantly lower in the DM+P group than in the C group at the 8-week follow-up (95% CI, −34.228 to −2.786; *p* = 0.017) (Figure 3A). No significant differences in the alternation rate were observed between the C and P groups or between the C and DM groups.

In the two-way repeated measures ANOVA, the main effect of time on the total number of arm entries was not significant, nor was the interaction between group and time. In Tukey’s post hoc test, the total number of arm entries did not differ significantly among the four groups at any time point (Figure 3B).

### 2.4. Comparison of Aβ Plaque Deposition

**Figure 4:**
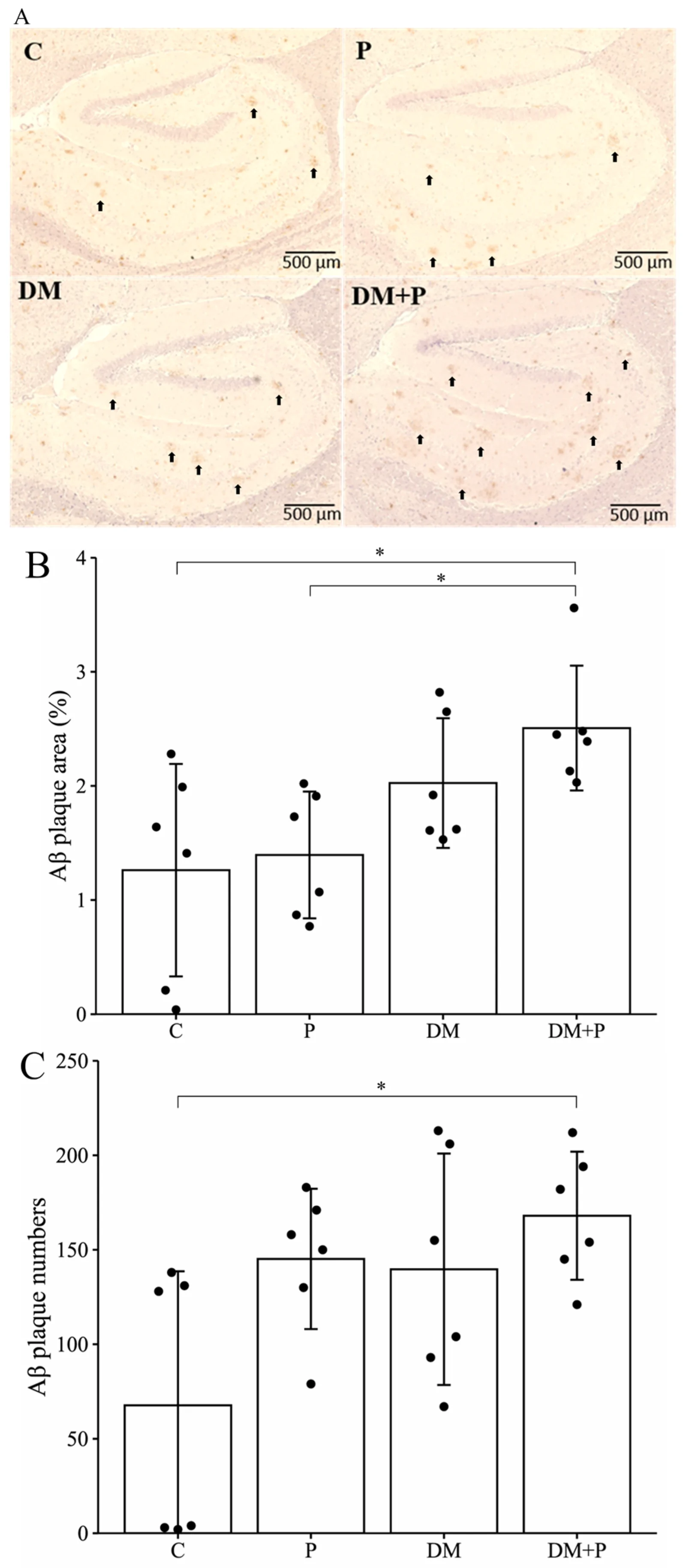
Comparison of Aβ plaque deposition. (**A**) Representative images of Aβ-immunostained hippocampal sections. Arrows indicate Aβ plaques. (**B**) Aβ plaque area. (**C**) Aβ plaque numbers. * *p* < 0.05. Data are expressed as the mean ± SD Aβ plaque area (%) and number of Aβ plaques in the hippocampus of mice in the C, P, DM, and DM+P groups. Statistical significance was determined using one-way ANOVA followed by Tukey’s post hoc test.

The percentage of Aβ plaque area in the hippocampus was significantly higher in the DM+P group than in the C (95% CI, 0.161 to 2.328; *p* = 0.021) and P (95% CI, 0.030 to 2.197; *p* = 0.043) groups (Figure 4A,B). The number of Aβ plaques was significantly higher in the DM+P group than in the C group (95% CI, 14.424 to 186.243; *p* = 0.019) (Figure 4C).

### 2.5. Comparison of the Number of Neurons

**Figure 5:**
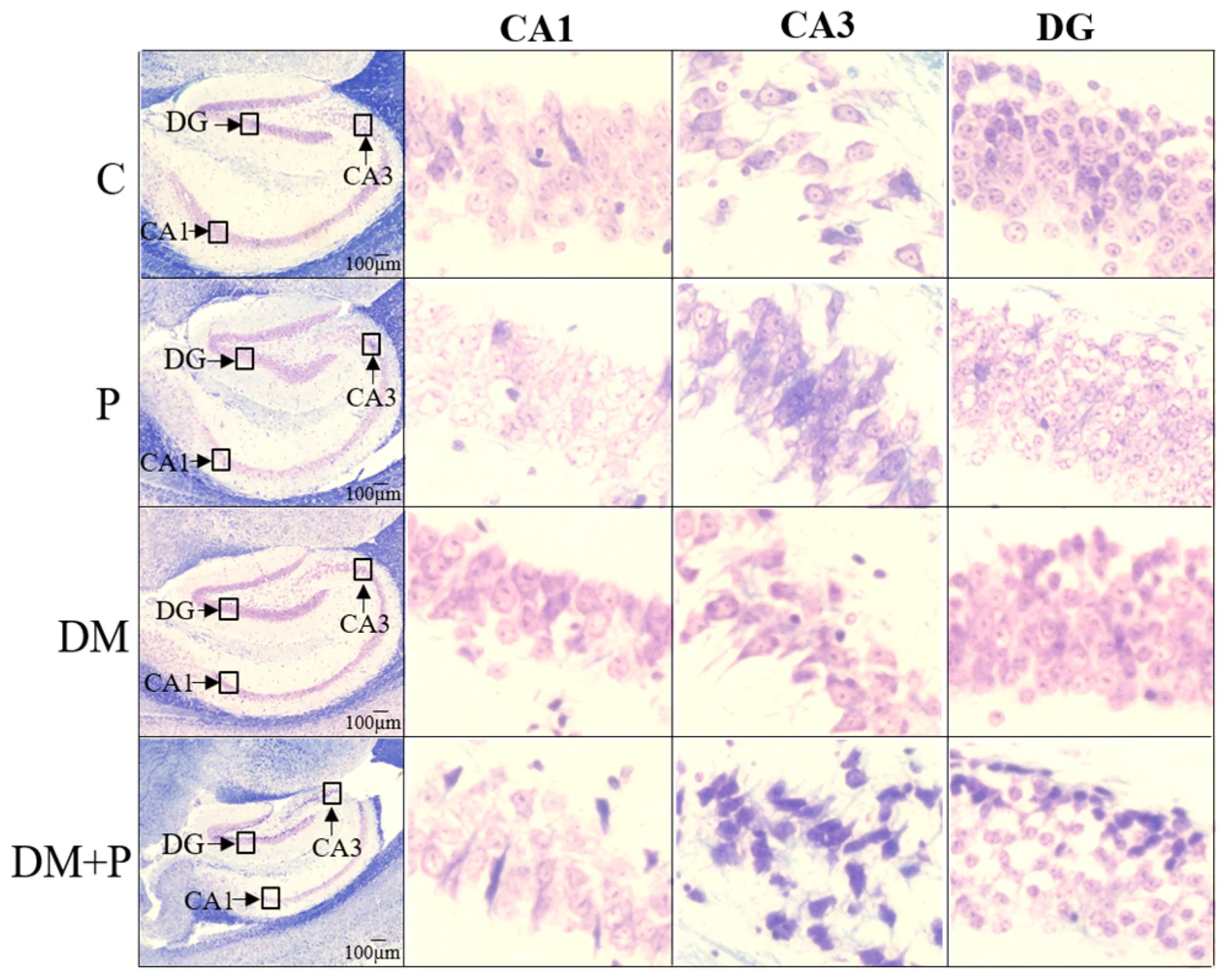
Analysis of the number of neurons using Klüver–Barrera staining. Representative images of the hippocampal CA1, CA3, and DG regions were cropped from the same section. Abbreviation: DG, dentate gyrus.

**Figure 6:**
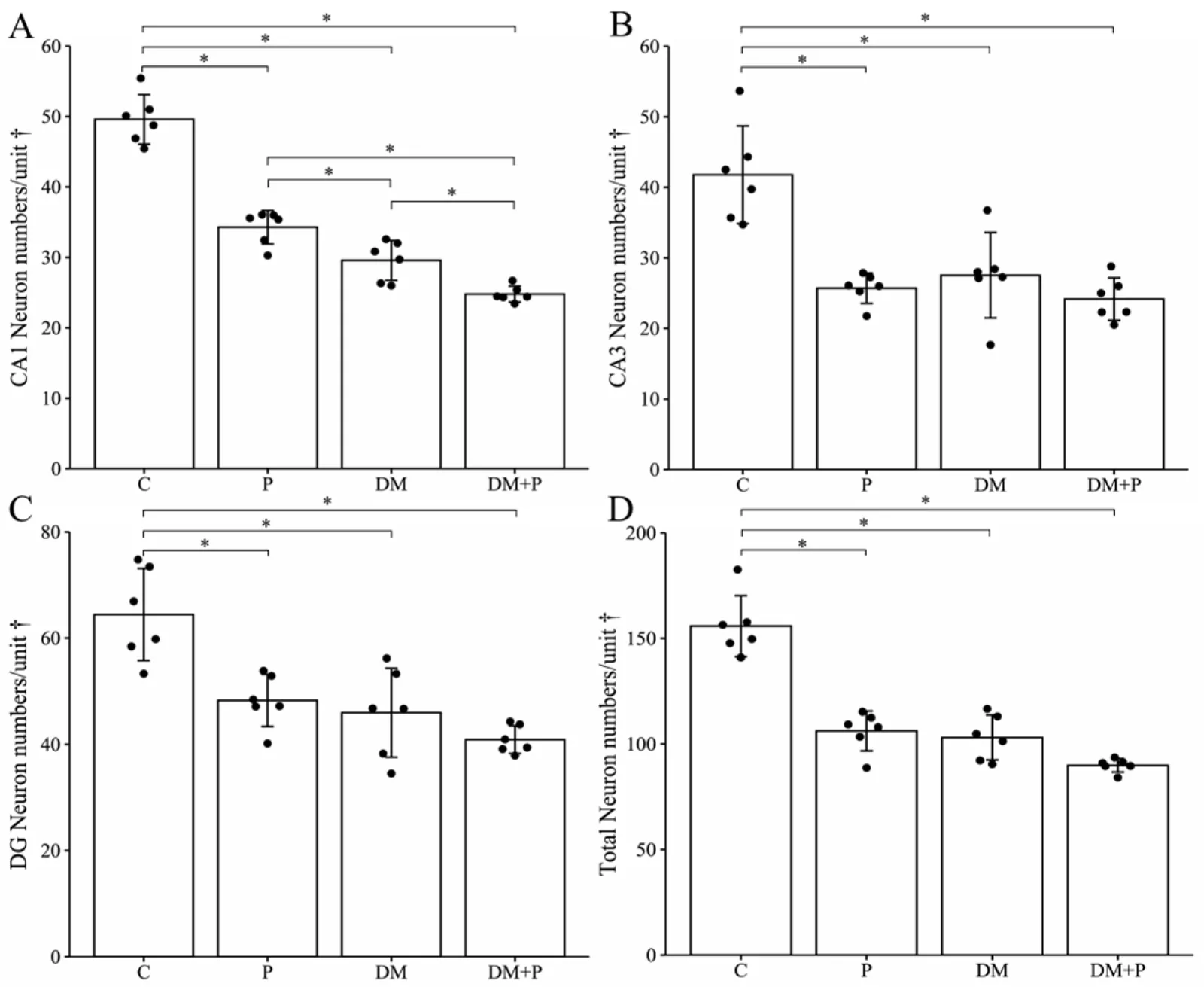
Comparison of the number of neurons per unit (100 × 100 µm^2^ area) in CA1, CA3, and the DG. (**A**) CA1 neuron numbers. (**B**) CA3 neuron numbers. (**C**) DG neuron numbers. (**D**) Total neuron numbers. * *p* < 0.05, † 100 × 100 µm^2^ area. Data are expressed as the mean ± SD number of neurons in CA1, CA3, and the DG of the hippocampus of mice in the C, P, DM, and DM+P groups. Statistical significance was determined using one-way ANOVA followed by Tukey’s post hoc test.

Figure 5 and Figure 6 show the results of a comparison of the number of neurons using Klüver–Barrera (KB) staining. Significantly fewer neurons were observed in CA1 in the P, DM, and DM+P groups than in the C group (95% CI, −19.534 to −11.097; *p* < 0.001; 95% CI, −24.251 to −15.814; *p* < 0.001; and 95% CI, −29.039 to −20.603; *p* < 0.001, respectively) (Figure 6A). The DM+P group also exhibited a significantly lower number of neurons than the P (95% CI, −13.724 to −5.287; *p* < 0.001) and DM (95% CI, −9.007 to −0.570; *p* = 0.023) groups. A significant difference was also noted between the P and DM groups (95% CI, 0.499 to 8.935; *p* = 0.025). In CA3, significantly fewer neurons were observed in the P, DM, and DM+P groups than in the C group (95% CI, −24.087 to −8.059; *p* < 0.001; 95% CI, −22.247 to −6.219; *p* < 0.001; and 95% CI, −25.636 to −9.609; *p* < 0.001, respectively) (Figure 6B). Significantly fewer neurons were observed in the dentate gyrus (DG) in the P, DM, and DM+P groups than in the C group (95% CI, −26.898 to −5.449; *p* = 0.002; 95% CI, −29.228 to −7.778; *p* = 0.001; and 95% CI, −34.280 to −12.830; *p* < 0.001, respectively) (Figure 6C). The total number of neurons was also significantly lower in the P, DM, and DM+P groups than in the C group (95% CI, −66.260 to −33.090; *p* < 0.001; 95% CI, −69.352 to −36.181; *p* < 0.001; and 95% CI, −82.565 to −49.394; *p* < 0.001, respectively) (Figure 6D).

### 2.6. miRNA and mRNA Expression

**Figure 7:**
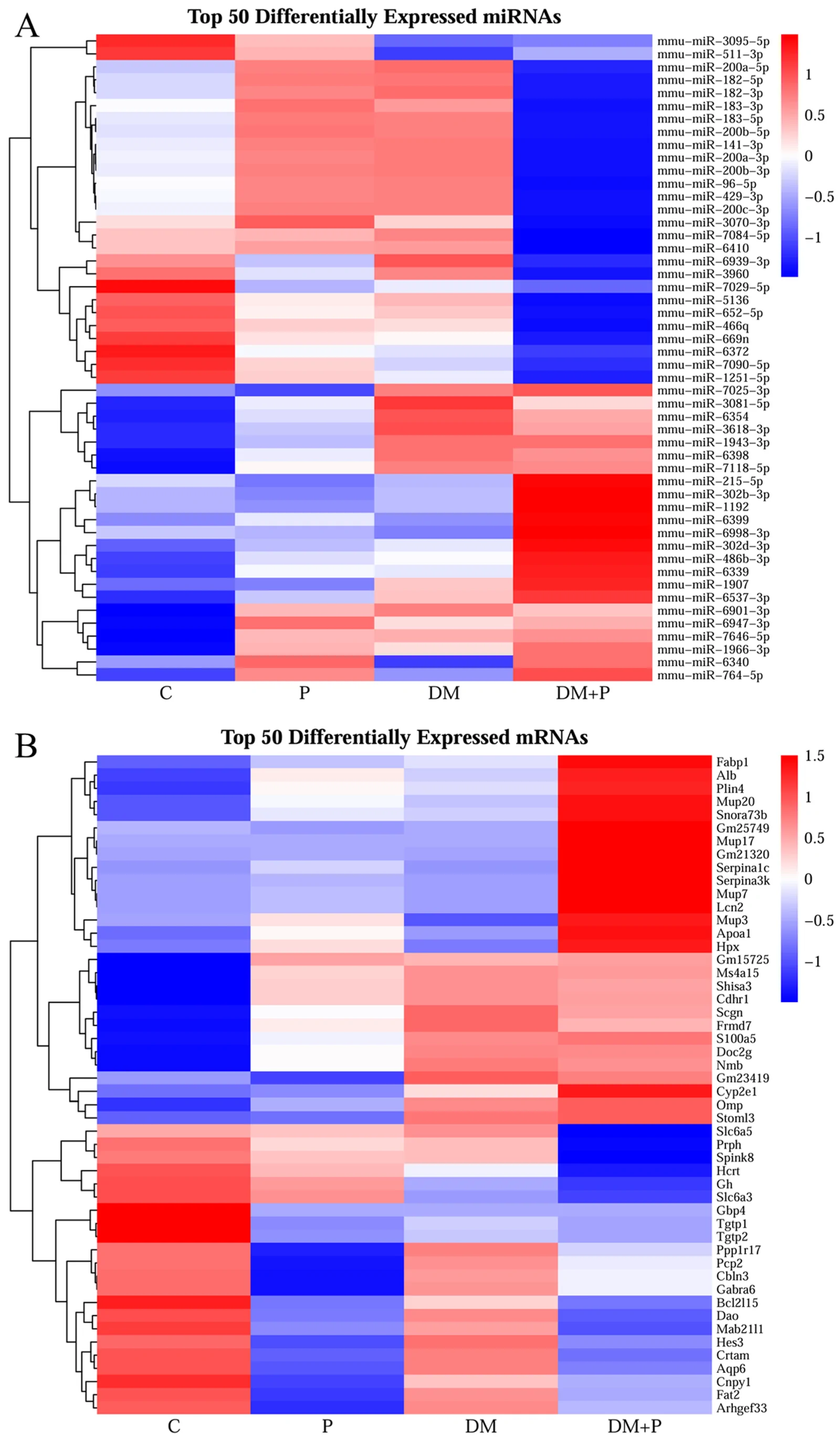
Heatmaps of the top 50 differentially expressed miRNAs and mRNAs between the C and DM+P groups. (**A**) Top 50 differentially expressed miRNAs. (**B**) Top 50 differentially expressed mRNAs. The heatmaps show expression profiles of the top 50 miRNAs and mRNAs ranked by the absolute log2 fold change in the DM+P group compared with the C group. Expression values were standardized using row-wise Z-scores. Red indicates relatively high expression, whereas blue indicates relatively low expression.

miRNA and mRNA expression was compared between the C and DM+P groups, between which significant differences had already been observed in most other measures. Because RNA samples from multiple mice were pooled prior to library preparation, the sequencing comparison was exploratory, and statistical testing was not performed for differential expression. Next-generation sequencing analysis identified 1466 mRNAs showing an absolute fold change (|FC|) ≥ 1.5 between the two groups. The complete mRNA and miRNA expression datasets, including raw read counts, normalized expression values, FC values, and annotation information, are provided in Supplementary Tables S1 and S2, respectively. Heatmaps showing the expression profiles of the top 50 differentially expressed miRNAs and mRNAs ranked by absolute log2 fold change (|log2FC|) were generated to visualize the sequencing results (Figure 7A,B).

Table 1 shows the association between miRNA and mRNA expression in the DM+P group compared with the C group in the integrated miRNA–mRNA analysis. Seven mRNAs were selected based on the integrated analysis. In the DM+P group, five mRNAs were downregulated (neuronal differentiation 1 [Neurod1], PR/SET domain 8 [Prdm8], BLM RecQ-like helicase [Blm], serine/threonine kinase 10 [Stk10], and neuronal PAS domain protein 3 [Npas3]), and two mRNAs were upregulated (T-box transcription factor 21 [Tbx21] and corneodesmosin [Cdsn]) with a |FC| > 1.5 compared with the C group. Among the seven identified mRNAs, Neurod1 showed the greatest reduction in expression. Neurod1 was predicted to be regulated by miR-693-3p, which was upregulated in the DM+P group compared with the C group (|FC| ≥ 1.5).

### 2.7. Neurod1 Expression in the Hippocampus

**Figure 8:**
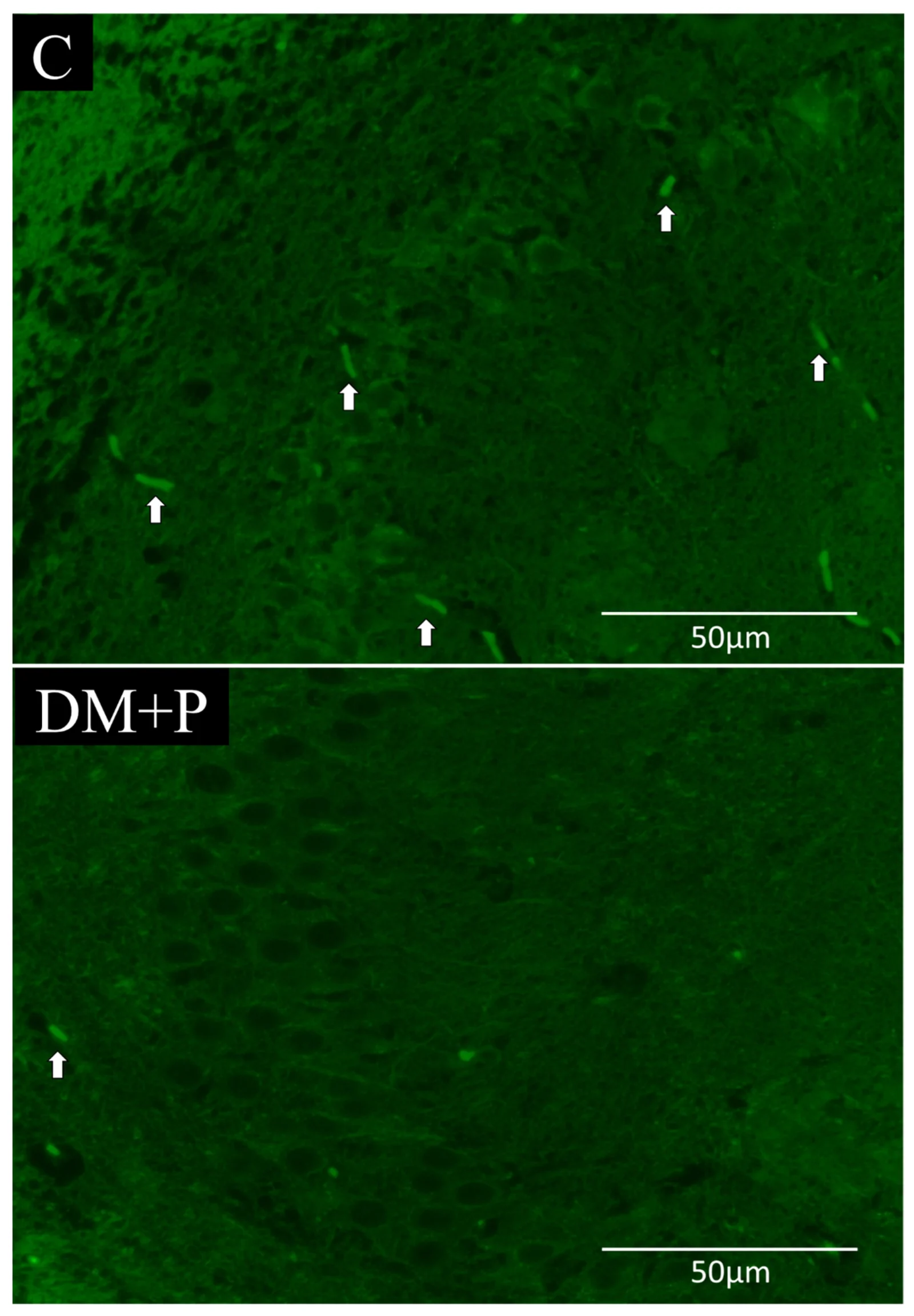
Neurod1 expression in the CA3 of the hippocampus in the C and DM+P groups, as determined by immunostaining using an anti-Neurod1 antibody. Arrows indicate Neurod1 expression.

**Figure 9:**
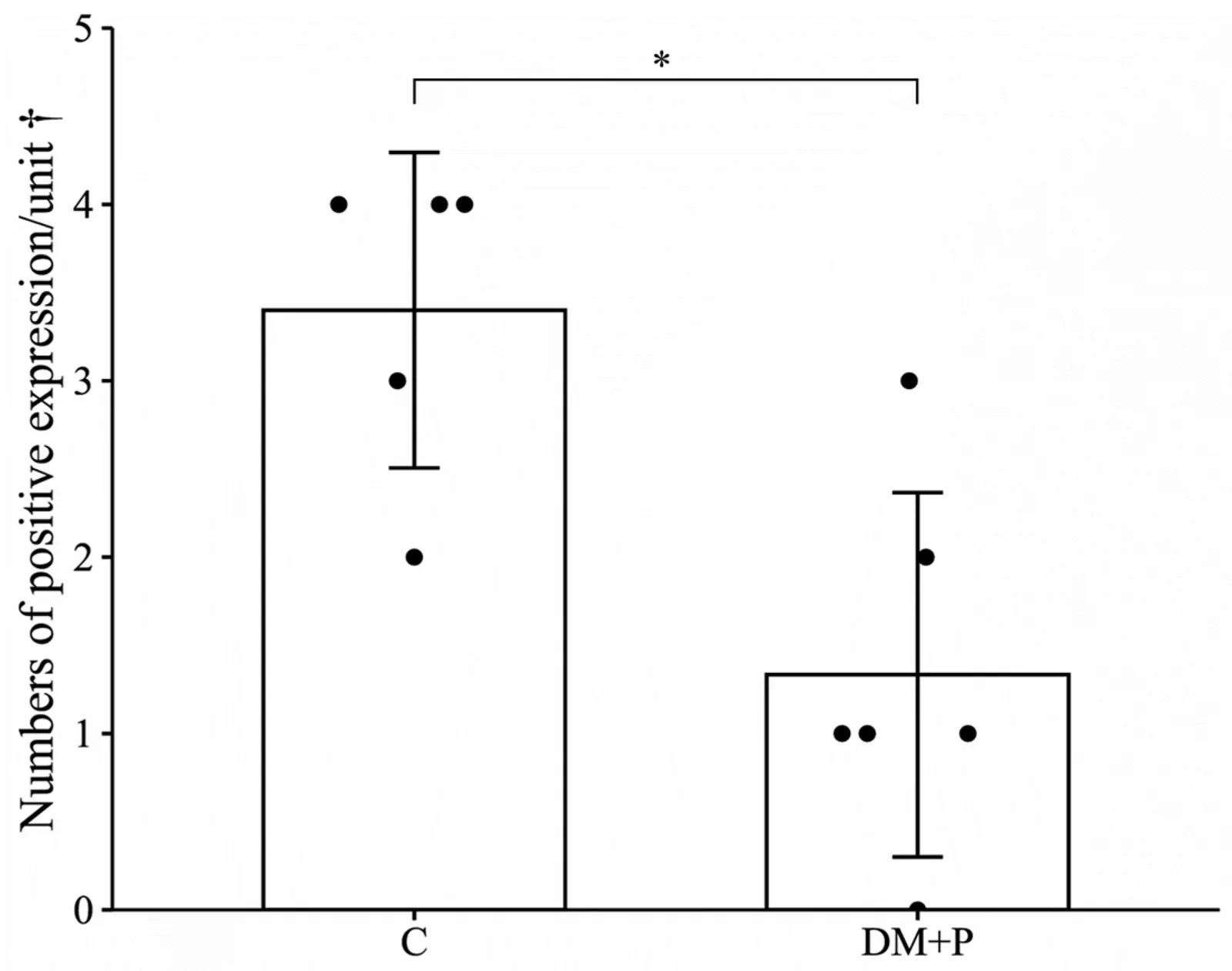
Comparison of Neurod1 expression in the CA3 of the hippocampus in the C and DM+P groups. * *p* = 0.006, † 100 × 100 µm^2^ area. Data are expressed as the mean ± SD of Neurod1 expression in the CA3 of the hippocampus per unit (100 × 100 µm^2^ area) in the C (*n* = 5) and DM+P (*n* = 6) groups. Statistical significance was determined using Welch’s *t*-test.

Based on the results of the integrated analysis, we focused on Neurod1, which showed the greatest reduction in expression among the seven identified mRNAs (|FC| = 4.10). Expression of Neurod1 protein in the CA3 of the hippocampus was significantly lower in the DM+P group than in the C group (95% CI, −3.382 to −0.751; *p* = 0.006; C, *n* = 5; DM+P, *n* = 6) (Figure 8 and Figure 9).

## 3. Discussion

The results of this study using AD model mice provide exploratory insights into the potential role of miRNA–mRNA regulatory relationships in AD-associated changes following combined exposure to periodontitis and DM. Mice in the DM+P group exhibited significantly greater cognitive impairment, increased cerebral Aβ deposition, and decreased neuronal numbers compared with the C group. The integrated analysis identified candidate miRNA–mRNA pairs based on predicted target relationships and inverse expression patterns between miRNAs and mRNAs. Overall, these findings suggest that the combined exposure to periodontitis and DM may be associated with AD-like pathological changes and alterations in miRNA and mRNA expression.

Alveolar bone loss was significantly greater in the P group than in the C and DM groups and in the DM+P group than in the C and DM groups. Our results showed that ligature placement in periodontal tissue leads to alveolar bone loss in the present AD model. This result was consistent with our previous study [48].

The alteration rate was significantly lower in the DM+P group than in the C group at 8 weeks. The percentage of Aβ deposit area in the hippocampus was significantly higher in the DM+P group than in the C and P groups. The number of Aβ deposits was significantly higher in the DM+P group than in the C group. In the Y-maze test, the total number of arm entries did not differ significantly among the four groups at any time point, suggesting that the observed reduction in alternation rate was unlikely to be attributable to differences in general locomotor activity. However, alternation rate may also be influenced by factors such as motivation, anxiety, and general health status, and these factors cannot be completely excluded in the present study [49,50]. Previous studies showed that ligature-induced periodontitis results in impairment of short- and long-term memory in 3× transgenic AD mice and increased levels of insoluble Aβ42 in 5× FAD mice [51,52]. Although different from ligature-induced periodontitis, AD model transgenic mice that developed *Porphyromonas gingivalis*-induced periodontitis showed a decrease in cognitive function, increased cerebral Aβ deposition, and enhanced intracerebral inflammatory responses [53,54]. In AβPP/PS1 double-transgenic mice treated with *P. gingivalis*–lipopolysaccharide (LPS) injection and ligation, spatial and non-spatial learning and memory were significantly decreased and Aβ deposition increased in the hippocampal region and cerebral cortex compared with control mice [55]. Several studies using wild-type mice reported that C57BL/6 mice infected with *P. gingivalis* or injected with *P. gingivalis*-derived LPS also exhibited cognitive impairment and deposition of Aβ in the hippocampus and cortex [56,57,58]. Of the periodontal models currently available, the ligature-induced periodontitis model is widely used because it closely reflects human periodontitis in promoting the accumulation of oral bacteria, leading to inflammation and subsequent periodontal bone destruction [52]. In addition, previous studies examining the effects of feeding AD mice a high-fat diet (HFD) suggested that HFD-fed AD model mice exhibit cognitive impairment and an increase in Aβ deposition in the cerebral cortex or hippocampus [59,60,61,62,63,64,65]. Taken together, these data and ours suggest that despite differences in periodontitis induction methods, our results are consistent with those of previous studies showing that both DM and periodontitis are associated with cognitive decline and brain Aβ deposition.

In the CA1, CA3, and DG regions of the hippocampus, the total number of neurons in the P, DM, and DM+P groups was significantly lower compared with the C group. The total number of neurons in the CA1 region was significantly lower in the DM+P group than in the P and DM groups. In AD model mice treated with *P. gingivalis*–LPS injection and ligation, the number of viable neurons in the cerebral cortex and the CA1 and DG regions of the hippocampus was significantly reduced due to atrophy or dissolution of Nissl bodies [55]. In addition, significantly fewer neurons were detected in the cortex and hippocampus regions of *P. gingivalis*-infected 5× FAD mice [53]. A previous study using C57BL/6 mice reported that mice orally inoculated with *P. gingivalis* or *Treponema denticola* had significantly fewer intact neuronal cells, higher numbers of dead cells in the hippocampus, and increased brain neuroinflammation than control mice [56,66]. In mice fed a HFD, hippocampal neurogenesis and neural progenitor cell proliferation were impaired [67,68,69,70]. In both insulin-resistant and insulin-deficient mice, DM impairs hippocampus-dependent memory and adult neurogenesis [71]. T2DM models using both obesity-independent MKR transgenic mice and obesity-dependent db/db mice showed compromised hippocampal neurogenesis [72]. These previous results support the findings of the present study. The CA1, CA3, and DG subregions of the hippocampus are involved in learning and memory [73,74]. The present results suggest that periodontitis and DM may contribute to hippocampal neuronal damage, which may be associated with impaired cognitive function [55].

Neurod1, identified through the integrated analysis, showed significantly lower protein expression in the hippocampal region in the DM+P group compared with the C group. Neurod1 is a proneural, basic helix-loop-helix transcription factor essential for proper development of the central nervous system, particularly in terms of the generation of granule cells in the hippocampus and cerebellum [75,76]. Neurod1 plays essential roles in terminal neuronal differentiation, maturation, and survival in both embryonic and adult neurogenesis [77,78]. Overexpression of Neurod1 is sufficient to promote neuronal differentiation of adult hippocampal neural progenitor cells, but deletion of Neurod1 results in reduced survival and maturation of new neurons [77,79]. The removal of Sry-related high-mobility-group box 2–dependent repression of Neurod1 via Wnt signaling is required for neurogenesis to proceed [80,81]. Retrovirus-induced overexpression of Neurod1 in the cortices of AD model mice resulted in reprogramming of reactive glial cells into functional glutamatergic neurons that generated spontaneous synaptic responses and induced postsynaptic responses [82]. Following astrocyte-to-neuron gene therapy with Neurod1, regenerated neurons can fire action potentials and integrate into pre-existing neural networks, ultimately improving cognition in AD mice [83]. Furthermore, loss of Neurod1 function results in severe DM, indicating that Neurod1 plays a critical role in functional development of the endocrine pancreas [84,85,86]. Neurod1 also plays an important role in the achievement and maintenance of β-cell maturation and function as well as in insulin transcription [84,87,88]. Neurod1 is also closely involved in increasing Ins2-induced brain-derived insulin secretion via the Wnt/β-catenin pathway [89,90]. The downregulated brain insulin associated with T2DM weakens insulin signaling activity and leads to tau hyperphosphorylation, which contributes to the development and progression of AD [91,92]. Therefore, the upregulation of miR-693-3p and downregulation of Neurod1 mRNA in the brain may be associated with alterations in neuronal differentiation, survival, maturation, and regeneration as well as brain insulin-related pathways, and may contribute to AD-related changes.

Prdm8 has been suggested to be required for the normal development of upper-layer neurons in the mammalian neocortex [93,94]. miR-302 has been identified as a critical and pleiotropic regulator of neuroepithelial differentiation during neurulation [95]. Additionally, miR-302b-3p was shown to be involved in neuronal apoptosis, and altered expression of miR-302b-3p has been associated with neuronal injury and oxidative stress [96,97]. Understanding of the relationship between DM, periodontitis, and Prdm8 is limited, but we postulated that the expression of miR-302d-3p, miR-302b-3p, and miR-466k is upregulated and that of Prdm8 is downregulated, which may be associated with altered neuronal differentiation and cognitive function.

Blm belongs to the RecQ helicase family and is associated with neuronal development [98]. Blm has also been suggested to be involved in the neuronal cell cycle re-entry observed in the AD-affected brain [99]. Although experimental evidence regarding the relationship between Blm and periodontitis is lacking, BLM has also been identified as an aging-related gene associated with periodontitis in a bioinformatics analysis [100]. Bloom syndrome is an autosomal recessive disorder caused by variants in the Blm gene, and complications include DM [101]. Both miR-29a and miR-29c are reportedly downregulated in the AD-affected brain [102,103,104,105]. However, the expression of miR-29a-3p, miR-29b-3p, and miR-29c-3p is consistently increased in different tissues in several metabolic conditions, including obesity, insulin resistance, and T2DM [106]. Rojas-Criollo et al. showed that overexpression of miR-29c-3p in HFD-fed mice exhibiting altered metabolic status (obesity and glucose derangements) could be interpreted as a counterregulatory mechanism against the deleterious effects of such a diet on the brain [107]. In addition, miR-29a-3p is reportedly associated with diabetic periodontitis [108]. Based on these data, we hypothesized that upregulated expression of miR-29a-3p and miR-29c-3p and downregulated expression of Blm are associated with the effects of DM and periodontitis in AD.

Stk10 is a member of the STE20 serine/threonine kinase family and plays an important role in tumor progression and may act as a tumor suppressor involved in apoptosis in tumor cells [109]. Stk10 overexpression was shown to inhibit apoptosis in spinal cord neuronal cells [110]. However, no association between Stk10 and AD, DM, or periodontitis can be inferred due to a lack of reported data.

Npas3 is a member of the basic helix-loop-helix family, a group of related proteins involved in hippocampal neurogenesis, hippocampal neuronal proliferation, and brain glucose metabolism [111,112,113,114]. Npas3-deficient mice display behavioral abnormalities that have been associated with schizophrenia, including hyperactivity, decreased learning and memory, and impaired social recognition [115,116]. Additionally, upregulation of blood-borne miR-122-5p is associated with AD and T2DM [117,118,119,120]. Previous studies have shown that miR-106a-5p and miR-216a-5p are associated with AD, but the details remain unclear and are partially contradictory to our results [118,121,122,123]. Although evidence of a relationship between DM, periodontitis, and Npas3 is unclear, we hypothesized that upregulation of miR-29a-3p, miR-29c-3p, miR-450b-5p, miR-302d-3p, miR-122-5p, miR-217-5p, miR-106a-5p, miR-876-5p, and miR-216a-5p and downregulation of Npas3 may affect hippocampal neurons and thereby be associated with impairments in cognitive function and memory.

Tbx21 upregulation in white blood cells and natural killer cell subsets may be associated with AD [124,125]. Previous studies have shown that Tbx21 may be associated with insulin sensitivity and chronic periodontitis [126,127]. Downregulation of miR-15b-5p in the blood has been associated with AD, and previous in vitro studies indicated a possible role for miR-15b-5p in the pathogenesis of AD [128,129]. Downregulation of miR-15b-5p in the blood has been associated with T2DM, and the Kcnq1ot1/miR-15b-5p/Ccnd1 and Ccnd2 axis could be involved in the pathogenesis of DM [130,131]. Previous studies also showed that miR-15a-5p is downregulated in the brain and blood of AD patients and upregulated in the gingiva and gingival crevicular fluid of those with periodontitis, which is partially consistent with and partially contradictory to our results [132,133,134,135,136]. Taken together, available evidence suggests that downregulation of miR-15b-5p and miR-15a-5p and upregulation of Tbx21 are associated with the effects of DM and periodontitis on AD, but the literature is limited, and details are incomplete.

Studies have suggested that Cdsn is involved in nervous system development and keratinization [137]. A comprehensive bioinformatics analysis aimed at identifying abnormally methylated differentially expressed genes related to chronic periodontitis identified Cdsn as a gene associated with chronic periodontitis [138]. Elevated BACE1 protein levels and decreased miR-339-5p levels have been observed in AD brain specimens [139]. Furthermore, miR-339-5p may contribute to the development of AD through targeting of BACE1 [140]. Overexpression of miR-182 was shown to promote axon outgrowth and dendrite branching-out in mouse cortical neurons, suggesting that miR-182 plays a role in neuronal maturation in the early stages of neuronal development [141]. miR-182 expression is decreased in the hippocampus of aged mice, and its overexpression helps improve cognition in these mice [142]. Furthermore, long noncoding RNAs related to periodontitis mutually inhibit miR-182 and regulate its target gene, forkhead box protein O1, thereby inhibiting the Wnt pathway and enhancing the osteogenic effect in periodontal mesenchymal stem cells [143]. Although the data regarding Cdsn are limited and the details of its underlying mechanism are thus unclear, the downregulation of miR-339-5p and miR-182-5p and upregulation of Cdsn may be associated with the effects of periodontitis on AD.

Accumulating evidence suggests that *P. gingivalis*-derived LPS activates inflammasome signaling involving caspase-4/11 and caspase-1, thereby promoting neuroinflammation and amyloid pathology [144,145,146]. Although these pathways were not investigated in the present study, inflammasome signaling may represent another potential mechanism contributing to AD pathology associated with DM and periodontitis.

This study had some limitations. First, we did not investigate the expression levels of miRNAs in serum, gingival tissues, pancreatic tissues, visceral adipose tissues, liver tissues, or skeletal muscle. Therefore, whether periodontitis and DM contributed to AD-related changes through the miRNAs identified in the present study remains unclear. A recent clinical study reported an association between serum miRNAs and gingival crevicular fluid miRNAs and postulated that gingival crevicular fluid miRNA reflects a change in the periodontal environment [147]. Our previous study found that periodontitis altered the profile of circulating miRNAs in a ligature-induced periodontitis rat model [48]. These findings raise the possibility that gingival-derived miRNAs may enter the circulation, and miRNAs altered by periodontitis and DM may potentially contribute to AD-related changes. Further investigations of miRNA expression levels in serum and gingival tissues are necessary to fully understand the effects of periodontitis and DM on AD.

Second, we only performed miRNA–mRNA pairing analysis, and the major miRNAs and target genes identified by sequencing were not validated by independent methods such as RT-qPCR. Although miR-693-3p was predicted to regulate Neurod1 by multiple target prediction databases, including PITA, microRNAorg, and TargetScan, the direct interaction between miR-693-3p and Neurod1 has not been experimentally validated. Further studies incorporating RT-qPCR validation and functional assays, such as luciferase reporter assays, will be needed to confirm these findings and obtain a more detailed understanding of the roles of these miRNAs.

Third, Aβ deposition and Neurod1 protein expression were evaluated only by immunohistochemistry without complementary biochemical validation, such as Western blot analysis. Future studies incorporating additional biochemical approaches will be important to further validate the present findings.

Fourth, inflammatory markers, glial activation markers, and inflammasome-related proteins were not evaluated in this study, and future studies incorporating these analyses are needed to further clarify the mechanisms linking periodontitis and DM to AD pathology.

Fifth, although fasting blood glucose levels were significantly higher in the DM and DM+P groups than in the C and P groups, other metabolic parameters, such as insulin levels, glucose tolerance, and insulin resistance, were not evaluated. Therefore, the metabolic phenotype of the DM and DM+P groups was not comprehensively characterized, and further metabolic assessments will be needed to better define the characteristics of this model.

Sixth, the sample size in each experimental group was relatively small (*n* = 6). Although an a priori sample size calculation was performed based on preliminary Y-maze test data, the possibility that some biologically relevant differences were not detected because of limited statistical power cannot be completely excluded. In addition, one mouse in the C group was excluded from the Neurod1 immunohistochemical analysis because of loss of the sample, resulting in *n* = 5 in the C group and *n* = 6 in the DM+P group. This further reduced the statistical power of this comparison, and the observed difference should therefore be interpreted with caution. In addition, because RNA samples from multiple mice were pooled within each experimental group before sequencing, statistical testing of the RNA-seq data was not performed. Future studies with larger sample sizes and individual-level sequencing analyses will be important to further validate the present findings.

Seventh, although the ligature-induced periodontitis model is widely used because it closely reflects human periodontitis, the possibility of mechanical damage from ligature insertion cannot be ruled out [52]. Insertion of ligatures may exacerbate periodontal tissue destruction and promote bone resorption. However, efforts were made to minimize any damage to the periodontium during surgery. Histological evaluation of gingival inflammation and assessment of bacterial burden were not performed to further confirm the induction of periodontitis.

Finally, all animals examined in this study were male. Different results may have been obtained in female mice. Clinical data indicate that women are not only more likely to develop AD than men but also to experience more rapid cognitive decline [148,149]. Most studies that have examined sex-related differences have reported that Aβ accumulation in brain tissue occurs earlier and progresses at an accelerated rate in female 3× Tg-AD mice [150]. A previous study suggested that age-related changes in the immune response may act as a key contributor to sex differences in AD trajectories [151]. Therefore, the generalizability of our findings to female animals remains unclear. However, further research is needed to elucidate the mechanisms leading to sex differences in AD.

## 4. Materials and Methods

### 4.1. Animals

Knock-in mice (B6-AppNL-G-F/NL-G-F/J) (6-month-old, male, 35.1 ± 3.2 g) were maintained in a 12 h light/dark cycle (22–25 °C temperature and 30–50% humidity) with free access to food and drinking water [152]. All animal experiments were approved by the Animal Care and Use Committee of Okayama University (Approval No. OKU-2018513) and followed the ARRIVE guidelines.

### 4.2. Experimental Design

The experimental period was 8 weeks. Twenty-four knock-in mice were used as the AD model [152]. An a priori sample size calculation was performed using preliminary Y-maze test data based on a one-way ANOVA comparing four groups (effect size, 1.07; alpha, 0.05; power, 0.80), and the estimated minimum sample size was four animals per group. Mice were allocated to four groups according to a predefined quasi-randomization sequence: (i) control (C) group (*n* = 6), (ii) DM group fed a high-fat/high-sucrose diet (DM) (*n* = 6), (iii) ligature-induced periodontitis group (P) (*n* = 6), and (iv) DM + periodontitis (DM+P) group (*n* = 6). During the experimental period, the C group was fed a normal diet (MF, Oriental Yeast Co., Ltd., Tokyo, Japan). The DM group was fed a high-fat/high-sucrose diet containing 58 kcal% fat from coconut oil and 28 kcal% sucrose (D12331, Research Diets, Inc., New Brunswick, NJ, USA) [153]. The P group was fed a normal diet. In the P group, a 5-0 black silk ligature was placed in the submarginal position on the mandibular first molars under intraperitoneal anesthesia (0.3 mg/kg medetomidine hydrochloride [Domitor^®^; Nippon Zenyaku Kogyo Co., Ltd., Fukushima, Japan], 4.0 mg/kg midazolam hydrochloride [Dormicum^®^; Astellas Pharma, Inc., Tokyo, Japan], 5.0 mg/kg butorphanol tartrate [Vetorphale^®^; Meiji Seika Pharma Co., Ltd., Tokyo, Japan]) to induce periodontitis [154]. The DM+P group was fed a high-fat/high-sucrose diet with induction of periodontitis. Mice in the control group were only anesthetized. Body weight measurements and the Y-maze test were performed at baseline, 4 weeks, and 8 weeks. Fasting blood glucose levels were measured at 8 weeks.

After the experimental period, all mice were euthanized under isoflurane inhalation. The brain from each mouse was extracted, and one portion was used for genetic analysis. The remaining portion was fixed overnight in 4% paraformaldehyde and then embedded in paraffin for histopathological evaluation. The dissected mandible was used for alveolar bone loss measurement. Investigators performing the Y-maze test, alveolar bone loss measurement, and histopathological evaluation were blinded to group allocation throughout the study.

### 4.3. Measurement of Alveolar Bone Loss

The right mandibular molar regions were de-fleshed with dental curettes and stained with 1% aqueous methylene blue solution (Sigma-Aldrich Co., LLC, St. Louis, MO, USA) for 5 min. A digital camera (Nikon Instruments Inc., Tokyo, Japan) was used to capture photographs of the stained mandible. The distance from the cement–enamel junction (CEJ) to the alveolar bone crest (ABC) of the first molar’s three roots was calculated (in mm) by image analysis using ImageJ software (version 1.53h, National Institutes of Health, Bethesda, MD, USA). The volume of alveolar bone loss was defined as the average of the values of the measurements at three points from the CEJ toward the ABC [155].

### 4.4. Assessment of Spatial Recognition Memory

Spatial recognition memory of the four groups of model mice was evaluated using the Y-maze test in a dark room at baseline and after 4 and 8 weeks [152,156]. The Y-maze apparatus consisted of three compartments (3 cm [W] bottom and 10 cm [W] top, 40 cm [L] and 12 cm [H]) radiating out from the center platform (3 × 3 × 3 cm triangle). Each mouse was placed in the center of the maze and allowed to freely explore the three arms for 8 min. An alternation was defined as entry into all three arms on consecutive choices (the maximum number of alternations was the total number of entries minus 2). The alternation rate was calculated as (actual alternations divided by maximum alternations) × 100 [157]. The total number of arm entries was also recorded as a measure of general locomotor activity. The alternation rate was designated as the spontaneous alternation behavior of the mice, and a high alternation rate indicated good memory function.

### 4.5. Quantification of Aβ Plaques and Neurons in the Hippocampus

Brain samples were cut into 4-µm-thick sections and subjected to immunohistochemical and KB staining. Accumulation of Aβ peptide plaques in the brain sample was evaluated by immunohistochemistry. Immunostaining for Aβ was performed based on the results of preliminary staining using a primary antibody against Aβ (anti-human amyloidβ [N] [82E1] mouse IgG MoAb, IBL, Gunma, Japan). The total number of Aβ plaque deposits and percentage of deposited area (%) within the selected visual field in the hippocampus were calculated. The number of neurons in the hippocampus was determined using KB staining. The number of neurons in each sample was determined by a single investigator (blinded to treatment) in the CA1, CA3, and DG regions and the total area of the hippocampus. The number of neurons within each 100 × 100 µm^2^ area was determined by dividing the total number of counted neurons by the number of applied areas. ImageJ software (National Institutes of Health) was used to measure Aβ plaque deposition and count neurons.

### 4.6. Total RNA Extraction

Brain samples from 6 mice per group were pooled prior to library preparation. Total RNA, including miRNAs, was extracted from brain samples using an RNA extraction kit (mirVana miRNA isolation kit, Thermo Fisher Scientific, Inc., Waltham, MA, USA). Total RNA samples were submitted to DNA Chip Research Inc. (Tokyo, Japan) for quality assessment and next-generation sequencing analyses. Extracted total RNA was quantified using a spectrophotometer (NanoDrop One, Thermo Fisher Scientific, Inc.). The quality of the extracted total RNA was evaluated using an Agilent 2100 Bioanalyzer (Agilent Technologies, Inc., Santa Clara, CA, USA) and associated reagent kit (Agilent RNA 6000 Nano kit, Agilent Technologies, Inc.).

### 4.7. Quantification of mRNA Expression Levels and Detection of Differentially Expressed Genes

Total RNA obtained from each sample was used for sequencing library construction using NEBNext Ultra II Directional RNA Library prep for Illumina (New England Biolabs, Ipswich, MA, USA) and NEBNext Poly(A) mRNA Magnetic Isolation Module (New England Biolabs), with NEBNext Multiplex Oligos for Illumina (96 Unique Dual Index Primer Pairs) (New England Biolabs). Library quality was assessed using an Agilent 2200 TapeStation (Agilent Technologies, Inc.) and Applied Biosystems 7500 Real-Time PCR system (Thermo Fisher Scientific, Inc.) with High Sensitivity D1000 reagents (Agilent Technologies, Inc.) and a GenNext NGS Library Quantification kit (Toyobo Co., Ltd., Osaka, Japan). The pooled libraries generated from the samples were sequenced using Illumina NextSeq 500 (Illumina, Inc., San Diego, CA, USA) with 75-base-pair (bp) single-end reads. The total number of original reads for the four groups ranged from 28.1 to 29.7 million per sample, and 99.8% of reads were retained after trimming.

Sequencing adaptors, low-quality reads, and bases were trimmed using the Trimmomatic-0.38 tool [158]. Sequence reads were aligned to the mouse reference genome (mm10) using STAR 2.7.8a [159]. Approximately 88.8–89.5% of reads were uniquely mapped to the reference genome. The aligned reads were analyzed using StrandNGS 4.0 software (Agilent Technologies, Inc.). Ensembl was used as the source for annotation [160,161]. Read counts for each gene and transcript were quantified and normalized using the transcripts per million method, which accounts for gene length and library size [160,162]. Because RNA samples from multiple mice were pooled prior to library preparation, statistical testing was not performed. Differentially expressed mRNAs were determined with an absolute value threshold of |FC| ≥ 1.5.

### 4.8. Quantification of miRNA Expression Levels and Detection of Differentially Expressed miRNAs

Sequencing libraries were constructed using a QIAseq miRNA Library kit (Qiagen, Hilden, Germany) and QIAseq miRNA NGS 96 Index IL (Qiagen) according to the manufacturer’s protocols. Library quality was assessed using an Agilent 2100 Bioanalyzer (Agilent Technologies, Inc.) and an Agilent High Sensitivity DNA kit (Agilent Technologies, Inc.). Pooled libraries were sequenced using Illumina NextSeq 500 (Illumina, Inc.) with 75 bp single-end reads. The total number of reads obtained for the four groups ranged from 10.0 to 11.4 million per sample. After adapter trimming and quality filtering, 94.1–94.7% of reads were retained for downstream analyses.

The QIAseq miRNA library kit utilizes the Unique Molecular Indexes (UMIs) system, which enables unbiased and accurate quantification of mature miRNAs. Original FASTQ files generated using NextSeq were uploaded to the Qiagen GeneGlobe Data Analysis Center (https://geneglobe.qiagen.com, accessed on 24 June 2021). The sequence reads were aligned to the mouse reference genome (mm10). The aligned reads were then deduplicated based on the UMIs and analyzed using StrandNGS v3.4 (Agilent Technologies, Inc.) and R v3.6.3 (R Foundation for Statistical Computing, Vienna, Austria) software. Read counts were normalized using the Trimmed Mean of M-value method, which adjusts for compositional differences between libraries [163]. MiRBase, release 21 (http://mirbase.org, accessed on 24 June 2021), was used as the source for annotation. Because RNA samples from multiple mice were pooled prior to library preparation, statistical testing was not performed. Differentially expressed miRNAs were determined with an absolute value threshold of |FC| ≥ 1.5.

### 4.9. Identification of mRNAs Targeted by miRNAs and Integrated miRNA–mRNA Analysis

Integrated miRNA–mRNA analysis was performed by predicting potential target mRNAs of these differentially expressed miRNAs using PITA, microRNAorg, and TargetScan [164,165,166]. mRNAs predicted by all three databases were considered potential targets. The predicted target mRNAs were compared with mRNA expression profiles, and candidate miRNA–mRNA pairs were selected based on an absolute mRNA |FC| ≥ 1.5 and inverse expression patterns between miRNAs and predicted target mRNAs. Heatmaps were generated using the top 50 differentially expressed miRNAs and mRNAs ranked by absolute |log2FC| to visualize expression patterns. Expression values for heatmap visualization were standardized using row-wise Z-scores.

### 4.10. Immunohistochemical Staining of the Protein Encoded by the Top Gene in the Integrated Analysis

We focused on the protein encoded by Neurod1, which showed the greatest reduction in expression among the identified mRNAs in the integrated analysis, and compared the expression of Neurod1 per unit area (100 × 100 µm^2^) in the CA3 of the hippocampus in each group using an anti-Neurod1 antibody (goat anti-rabbit IgG H&L [Alexa Fluor^®^ 488, Cat# ab150077, 1:1000 dilution], Abcam, Cambridge, UK; Anti-Neurod1 antibody [EPR20766, Cat# ab213725, 1:1000 dilution], Abcam, Cambridge, UK). Due to damage caused during the preparation and staining of tissue sections, the number of samples in the C group was reduced from 6 to 5 for comparison of immunohistochemical staining results.

### 4.11. Statistical Analyses

Data are presented as the mean ± standard deviation (SD). One-way ANOVA followed by Tukey’s post hoc test was used for statistical comparisons of fasting blood glucose levels, alveolar bone loss, Aβ plaque deposition, and the number of neurons in the hippocampus. Two-way repeated measures ANOVA followed by Tukey’s post hoc test was used for statistical comparisons of body weight, the alternation rate, and the total number of arm entries. These analyses were performed assuming normally distributed data. Homogeneity of variances was assessed using Levene’s test. Comparisons of Neurod1 immunoreactivity between the C and DM+P groups were performed using Welch’s *t*-test. Statistical analyses were performed using SPSS (v. 25.0; SPSS Inc., Chicago, IL, USA). In all analyses, *p* < 0.05 was taken to indicate statistical significance.

## 5. Conclusions

The results of the present study suggest that the combined effects of periodontitis and DM may be associated with AD-like pathological changes. Neurod1 expression was suppressed at both the gene and protein levels, and the exploratory integrated miRNA–mRNA analysis suggested that miR-693-3p may regulate Neurod1. These findings identify the miR-693-3p/Neurod1 pair as a candidate regulatory axis potentially involved in the association of periodontitis and DM with AD-like pathological changes.

## Figures and Tables

**Table 1:** Candidate miRNA–mRNA pairs identified in the integrated analysis based on predicted target relationships and inverse expression patterns between the C and DM+P groups.

miRNA		mRNA		Full Name	Absolute FC
Upregulated	miR-693-3p	Downregulated	Neurod1	neuronal differentiation 1	4.10
	miR-302d-3p, miR-302b-3p, miR-466k		Prdm8	PR/SET domain 8	1.82
	miR-29a-3p, miR-29c-3p		Blm	BLM RecQ-like helicase	1.60
	miR-449c-5p, miR-450b-5p, miR-452-5p		Stk10	serine/threonine kinase 10	1.51
	miR-29a-3p, miR-29c-3p, miR-450b-5p, miR-302d-3p, miR-122-5p, miR-217-5p, miR-106a-5p, miR-876-5p, miR-216a-5p		Npas3	neuronal PAS domain protein 3	1.64
Downregulated	miR-15b-5p, miR-15a-5p	Upregulated	Tbx21	T-box transcription factor 21	4.20
	miR-339-5p, miR-182-5p		Cdsn	corneodesmosin	1.56

Absolute FC, absolute fold change in mRNA for the DM+P group using the C group as reference. RNA samples from multiple mice were pooled prior to library preparation; therefore, statistical testing was not performed.

## Data Availability

The raw sequencing data generated in this study have been deposited in the NCBI Gene Expression Omnibus (GEO) under accession numbers GSE344050 for RNA-seq and GSE344051 for miRNA-seq.

## Supplementary Materials

The following supporting information can be downloaded at: https://www.mdpi.com/article/10.3390/ijms27177542/s1.
